# *In silico *identification of functional divergence between the multiple *groEL *gene paralogs in *Chlamydiae*

**DOI:** 10.1186/1471-2148-7-81

**Published:** 2007-05-22

**Authors:** David McNally, Mario A Fares

**Affiliations:** 1 Evolutionary Genetics and Bioinformatics Laboratory, Department of Genetics, Smurfit Institute of Genetics, University of Dublin, Trinity College, Dublin, Ireland

## Abstract

**Background:**

Heat-shock proteins are specialized molecules performing different and essential roles in the cell including protein degradation, folding and trafficking. GroEL is a 60 Kda heat-shock protein ubiquitous in bacteria and has been regarded as an important molecule implicated in chronic inflammatory processes caused by *Chlamydiae *infections. GroEL in *Chlamydiae *became duplicated at the origin of the *Chlamydiae *lineage presenting three distinct molecular chaperones, namely the original protein GroEL1 (Ct110), and its paralogous proteins GroEL2 (Ct604) and GroEL3 (Ct755). These chaperones present differential and independent expressions during the different stages of *Chlamydiae *infections and have been suggested to present differential physiological and regulatory roles.

**Results:**

In this comprehensive *in silico *study we show that GroEL protein paralogs have diverged functionally after the different gene duplication events and that this divergence has occurred mainly between GroEL3 and GroEL1. GroEL2 presents an intermediate functional divergence pattern from GroEL1. Our results point to the different protein-protein interaction patterns between GroEL paralogs and known GroEL protein clients supporting their functional divergence after *groEL *gene duplication. Analysis of selective constraints identifies periods of adaptive evolution after gene duplication that led to the fixation of amino acid replacements in GroEL protein domains involved in the interaction with GroEL protein clients.

**Conclusion:**

We demonstrate that GroEL protein copies in *Chlamydiae *species have diverged functionally after the gene duplication events. We also show that functional divergence has occurred in important functional regions of these GroEL proteins and that very probably have affected the ancestral GroEL regulatory role and protein-protein interaction patterns with GroEL client proteins. Most of the amino acid replacements that have affected interaction with protein clients and that were responsible for the functional divergence between GroEL paralogs were fixed by adaptive evolution after the *groEL *gene duplication events.

## Background

Cells use several mechanisms to ameliorate the effects of transient changes in the environmental conditions such as heat stress, irradiation, viral infections, etc. For instance, cells have developed a complex family of genes coding for protein-folding machines sharing a wide range of vital functions to buffer the effects of stress on the proteome integrity. These proteins, also called heat-shock proteins or molecular chaperones, are classified in different protein families named on the basis of their members' approximate molecular weight and they assist in the folding, trafficking and degradation of proteins [1-3]. The heat-shock protein GroEL is among the best-studied molecular chaperones in bacteria and belongs to the group I chaperonins. Group I chaperonins are a group of ring-shaped ATPases that assist *de novo *protein folding in most cellular compartments [4-8]. GroEL is a homotetradecamer that interacts with a ring-shaped cofactor named GroES, which participates in folding proteins into the correct three-dimensional conformation [9,10], and both proteins are essential for *Escherichia coli *growth at all range temperatures [11].

Due to the important functional role played by GroEL in maintaining the proteome integrity of cells, GroEL has become the target of many microbiological studies aimed at uncovering molecules involved in the epidemiology of pathogenic bacteria. GroEL from pathogenic bacteria is a highly immunoadjuvant protein and is recognised by the Toll-like receptors as part of the innate defence system [12,13]. The fact that GroEL is among the most conserved protein families [13] and that GroEL isolated from pathogenic bacteria has been reported to have a strong immune eliciting function [14] has inspired projects aimed at developing vaccines targeting GroEL from pathogens. These studies yielded insightful results implicating GroEL in bacterial disease pathogenesis such as those caused by *Chlamydiae *infections [15]. GroEL in *Chlamydiae trachomatis *(also called Ct110) has been implicated in chronic inflammatory processes caused by *Chlamydiae *infections leading to tissue damage and scarring [16-19]. Interestingly, GroEL in *Chlamydiae *became duplicated at the origin of the *Chlamydiae *lineage presenting three distinct molecular chaperones, namely the original protein GroEL1 (Ct110), and its paralogous proteins GroEL2 (Ct604) and GroEL3 (Ct755) [15]. Even though the three *Chlamydiae *GroEL proteins present substantial amino acid sequence conservation in important regions involved in polypeptide binding when compared to GroEL from the bacterium *Escherichia coli*, significant differences have been spotted in GroES binding regions and at regions involved in ATP binding and hydrolysis. Among the three *groEL *genes, only the expression levels of *groEL1 *and its cochaperone *groES *increase under heat-stress conditions and only the protein GroEL1 complements the function of a GroEL thermo-sensitive mutation in HeLa cells under heat-stress conditions [15]. Further, a previous report identified differences in the expression levels between the three *groEL *genes during the developmental stages of *C. trachomatis *[20]. This study also showed through *in vitro *models of *C. trachomatis *infection that the three different *groEL *genes are differentially and independently expressed during the different infection cycles of this pathogen, with *groEL2 *being highly expressed during the infectious cycle of *Chlamydiae *and *groEL3 *showing the highest expression among the three *groEL *genes during the persistent infections [20].

Despite previous efforts invested in unravelling the main functional differences between the three different *groEL *genes in *Chlamydiae*, results have brought more questions than they have answered regarding the reasons for this functional divergence. To date, apart from one study in 2003 conducting some computational analyses for these genes [15], no detailed bioinformatics approach has been performed to aid in understanding the evolutionary dynamic differences between the three *groEL *genes and to link these differences with functional data.

In this study we conduct state-of-the-art bioinformatics analyses to unravel the main selective constraints leading to the functional differentiation between the *Chlamydiae groEL *genes. To identify functional divergence between the different GroEL protein copies we test the selective constraints after *groEL *gene duplication, analyze and phylogenetically map amino acid sites involved in this functional divergence and conduct molecular coevolution analyses within GroEL proteins and between these and proteins known to be obligate *E. coli *GroEL protein clients. The effects of amino acid sites involved in functional divergence in the stability of GroEL protein structures are also discussed.

## Results

### GroEL proteins have diverged functionally in Chlamydiae after gene duplication

To test functional divergence between GroEL proteins after gene duplication we applied the program Diverge version 2.0 (See methods for details). Diverge tests for the presence of functional divergence of two types, functional divergence type I and type II. Functional divergence type I is detected when sites conserved (for example, showing no or low number of amino acid replacements when comparing sequences at that particular site) in one of the phylogenetic clusters (protein paralog) are significantly variable in the other related phylogenetic cluster. In other words, functional divergence type I indicates strong selective (and therefore functional) constraints at that site (for example, due to the acquisition or pre-existence of a functional role for that site) in one of the clusters and relaxed constraints (due to the loss or inexistent functional role at that site) in the paralogous cluster. Functional divergence type II is detected when, after gene duplication mutations leading to different amino acids become fixed in both resulting paralogous proteins and these mutations remain conserved after speciation in each cluster. This pattern indicates that amino acid sites diverged functionally between both paralogous clades (showing two distinct amino acids when comparing the two clades) but they were equally important for the protein's function (for example the amino acid remain conserved in each phylogenetic clade). We were interested in testing functional divergence type I to detect loss or acquisition of functional roles in particular amino acid sites in one of the GroEL group paralogs.

We subjected the multiple sequence alignments including the three GroEL protein paralogs to phylogenetic analyses and used the resulting phylogeny as an initial tree for the functional divergence test. In all the comparisons performed, the hypothesis of functional divergence provided significantly better log-likelihood values than the null hypothesis that assumes no functional divergence. In fact, GroEL1 showed functional divergence type I when compared to GroEL2 (θ = 0.371 ± 0.096; LRT = 15.025; *P *< 0.001) and GroEL3 (θ = 0.943 ± 0.099; LRT = 90.978; *P *<< 0.001). Interestingly, the parameter of functional divergence as well as the LRT was more significant when comparing GroEL1 to GroEL3 than in the case of the comparison of GroEL1 to GroEL2 (Table 1). GroEL2 showed moderate functional divergence from GroEL1 and stronger divergence from GroEL3 than from GroEL1 (θ = 0.441 ± 0.073; LRT = 36.014; *P *< 0.001). Comparison of GroEL1 to the cluster formed by GroEL2 and GroEL3 also yielded significant results (θ = 0.414 ± 0.117; LRT = 12.484; *P *< 0.001).

**Table 1:** Functional divergence type I analysis between GroEL protein paralogs in *Chlamydiae *species.

**Comparison**	**θ^a ^± SE(θ)**	**α^b^**	**LRT^c^**	***P*(LRT)**
GroEL1 vs GroEL2	0.371 ± 0.096	1.834	15.025	< 0.001
GroEL1 vs GroEL3	0.943 ± 0.099	1.921	90.978	<< 0.001
GroEL1 vs GroEL2-3	0.414 ± 0.117	3.448	12.484	< 0.001
GroEL2 vs GroEL3	0.441 ± 0.073	3.105	36.014	<< 0.001

^a ^The coefficient of Functional divergence type I calculated by maximum likelihood.

^b ^The shape parameter of the Gamma distribution of substitution rates among sites.

^c ^The likelihood ratio test to compare the likelihood of the hypothesis indicating no functional divergence to the hypothesis assuming functional divergence. LRT has been approached to a χ^2 ^distribution with 1 degree of freedom.

Functional divergence data is therefore in agreement with the expression divergence shown in previous functional/expression analysis demonstrating that in fact the different *groEL *gene copies are differently and independently expressed over time post-infection during *Chlamydiae *infection, and that GroEL3 is the most abundant protein at all time points assessed during the developmental cycle [20]. In their study however, GroEL3 was virtually absent during persistent infections and GroEL2 showed the highest expression levels at that stage [20]. Our results also support, in addition to the differential expression of the different *groEL *genes, the divergence in the protein function between the three *Chlamydiae *GroEL proteins.

**Figure 1:**
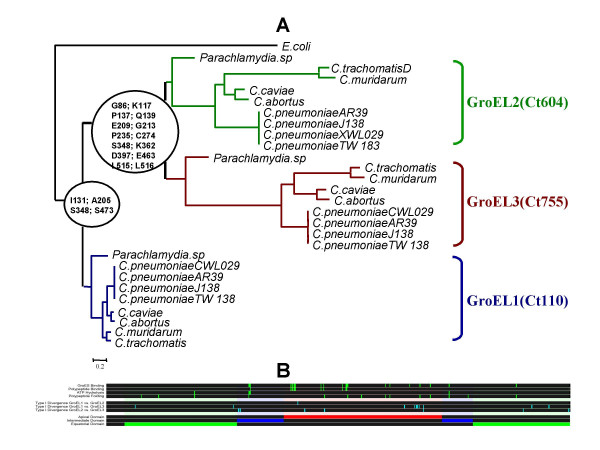
**Analysis of functional divergence type I in the multiple sequence alignment comprising sequence from GroEL1 (Ct110), GroEL2 (Ct604) and GroEL3 (Ct755) in *Chlamydiae***. A) The different GroEL paralogs are indicated and the sites detected with high posterior probabilities within the class of functional divergence type I are shown in each node corresponding to the gene duplication events. B) Distribution of selective constraints along the GroEL multiple sequence alignment. GroES binding, polypeptide binding, ATP hydrolysis and polypeptide folding domains are indicated as green bars in the first block. Sites under functional divergence in each one of the comparisons are indicated as blue bars in the second block. Apical, equatorial and intermediate domains are labelled in red, green and blue colours, respectively in the third block.

The difference in the magnitude of functional divergence between GroEL proteins can also be quantified by the identification of sites responsible for such functional divergence after each duplication event (Figure 1A). Examination of the distribution of sites under functional divergence (Figure 1B) when we compared GroEL1 to GroEL2 only yielded three amino acid positions under functional divergence using the posterior probability (PP) threshold of PP= 0.75. These sites were I131, A205 and E338 (Here we take the GroEL1 sequence of *Chlamydiae trachomatis*, with accession number: NP_219613 as the reference protein sequence). The homologous site of A205 in *E. coli *(C205) is located in a region involved in binding protein substrates [21]. In addition, E338 is the homologous position to A339 in *E. coli*, closely located to charged residues exposed to the central cavity in the *cis *GroEL ring, probably in contact with substrates [13]. These sites were conserved in GroEL1 but became variable in GroEL2, suggesting a loss of functional role in GroEL2 at these sites. Comparison of GroEL1 to the cluster formed by the paralogs GroEL2 and GroEL3 identified as sites significantly responsible for functional divergence type I I131, A205, S348 and S473 (Figure 1A and 1B). Apart from the obvious functional role of A205, S473 is the homologous position of G471 in *E. coli *and physically proximal to *E. coli *478–481 possition involved in ATP binding and hydrolysis [21]. Once again, these positions are highly conserved in GroEL1 and very variable in the cluster formed by the paralogs GroEL2 and GroEL3.

Comparison of GroEL1 to GroEL3 and GroEL2 to GroEL3 showed a great percentage of sites under functional divergence type I with threshold posterior probability values of PP = 0.75 and PP = 0.95 (Figure 1B). The number of sites detected was greater in the comparison of GroEL1 to GroEL3 than in GroEL2 to GroEL3 comparison. We also studied the pattern of functional divergence and found three different profiles represented by the amino acid sites under functional divergence. The first pattern presented sites conserved for GroEL1 and GroEL2 but variable for GroEL3 (supporting loss of functional constraints at that site in GroEL3) and was represented by 42.18% of the functionally divergent sites. The second pattern was that represented by sites (23.44%) that were variable in GroEL1 and GroEL2 and became conserved in GroEL3 (indicating a gain of functional constraints in GroEL3 as the most parsimonious hypothesis). Finally we also found sites (33.6% of sites) variable in GroEL1 that became conserved in GroEL2 and GroEL3 (indicating the possible loss of functional constraints at that site in GroEL1). In most of the cases hence, GroEL3 presented loss of functional constraints in some sites and gain of constraints in others and these results were more obvious for GroEL3 than for GroEL2 compared to GroEL1. Examination of the sites under functional constraints in GroEL3 provided evidence supporting the involvement of these sites in ATP binding (G86, homologous to G86 in *E. coli*), substrate and GroES binding (P235, homologous to P236 in *E. coli*) [21] and interaction and folding of protein clients in the GroEL central cavity (K362 and D397 homologous to K363 and D397, respectively in *E. coli*) [13]. These results suggest that functional divergence might have affected the interaction mainly between GroEL3 and its protein clients and to a lesser extent between GroEL2 and GroEL protein clients.

We also examined the effect that mutations between the different GroEL protein copies have on the protein structure. We modeled three-dimensional structures for GroEL1, GroEL2 and GroEL3 by homology to *Escherichia coli *GroEL protein using the program 3D-JIGSAW [22-24]. We then compared these structures using the Root Mean Square Deviation (RMSD) of the different atoms calculated as:

RMSD=1N∑i=1Ndi2
 MathType@MTEF@5@5@+=feaafiart1ev1aaatCvAUfKttLearuWrP9MDH5MBPbIqV92AaeXatLxBI9gBaebbnrfifHhDYfgasaacH8akY=wiFfYdH8Gipec8Eeeu0xXdbba9frFj0=OqFfea0dXdd9vqai=hGuQ8kuc9pgc9s8qqaq=dirpe0xb9q8qiLsFr0=vr0=vr0dc8meaabaqaciaacaGaaeqabaqabeGadaaakeaacqWGsbGucqWGnbqtcqWGtbWucqWGebarcqGH9aqpdaGcaaqaamaalaaabaGaeGymaedabaGaemOta4eaamaaqahabaGaemizaq2aa0baaSqaaiabdMgaPbqaaiabikdaYaaaaeaacqWGPbqAcqGH9aqpcqaIXaqmaeaacqWGobGta0GaeyyeIuoaaSqabaaaaa@3EFA@

Here, d is the atomic distance and N is the total number of atoms in the protein crystal. So for the comparison between proteins A and B, d will equal:

di=(Xai−Xbi)2+(Yai−Ybi)2+(Zai−Zbi)2
 MathType@MTEF@5@5@+=feaafiart1ev1aaatCvAUfKttLearuWrP9MDH5MBPbIqV92AaeXatLxBI9gBaebbnrfifHhDYfgasaacH8akY=wiFfYdH8Gipec8Eeeu0xXdbba9frFj0=OqFfea0dXdd9vqai=hGuQ8kuc9pgc9s8qqaq=dirpe0xb9q8qiLsFr0=vr0=vr0dc8meaabaqaciaacaGaaeqabaqabeGadaaakeaacqWGKbazdaWgaaWcbaGaemyAaKgabeaakiabg2da9maakaaabaWaaeWaaeaacqWGybawdaWgaaWcbaGaemyyaeMaemyAaKgabeaakiabgkHiTiabdIfaynaaBaaaleaacqWGIbGycqWGPbqAaeqaaaGccaGLOaGaayzkaaWaaWbaaSqabeaacqaIYaGmaaGccqGHRaWkdaqadaqaaiabdMfaznaaBaaaleaacqWGHbqycqWGPbqAaeqaaOGaeyOeI0IaemywaK1aaSbaaSqaaiabdkgaIjabdMgaPbqabaaakiaawIcacaGLPaaadaahaaWcbeqaaiabikdaYaaakiabgUcaRmaabmaabaGaemOwaO1aaSbaaSqaaiabdggaHjabdMgaPbqabaGccqGHsislcqWGAbGwdaWgaaWcbaGaemOyaiMaemyAaKgabeaaaOGaayjkaiaawMcaamaaCaaaleqabaGaeGOmaidaaaqabaaaaa@55CB@

Here, we are comparing the mean distance between amino acids a and b belonging to proteins A and B respectively by comparing their coordinates in the three space axes. This comparison did not detect any significant structural differences among the three GroEL proteins or between them and *E. coli *GroEL protein (The distances were all below 3.5Å). Results then suggest that amino acid replacements did not involve structural changes but rather may have induced functional shifts between GroEL protein copies.

Although no major structural changes seem to be related to sites under functional constraints we examined whether sites with varying degrees of selective constraints in the different GroEL copies show differences in the folding energy of the local GroEL structures. The performance of different methods to analyze local folding energies has been recently elegantly examined [25]. In their work, Rastogi et al., tested the accuracy of different models to predict the most stable structure or folding for four sets of proteins, Globin-like, SH3 domain, SH2 domain and Flavodoxin-like proteins. We used this methodology to look at folding-energy related differences at those sites under different functional constraints when comparing GroEL copies (for example, highly constrained amino acid sites in one GroEL copy but showing lack of constraints at another GroEL protein copy) and estimated the significance of these differences. We calculated this significance by comparing our folding-energy results with a distribution of folding energies for a 1000 randomly generated set of peptides sharing the same length and composition as the local fold of our proteins. We did the analyses using scripts and programs kindly provided by the group of Prof. Liberles. Our comparisons showed no significant differences in those sites under functional divergence when comparing the different mutant versions of the protein at those sites. In conclusion hence the mutations under varying functional constraints between GroEL copies lineages did not show significant variability in the local folding energies (Data not shown). Although apparently negative, the examination of the effects of mutations on protein structures is anything but straightforward. The reason is that two main factors have to be considered in such analyses. First, structures and folds are very flexible to mutations [26-28] and slight changes on function do not have to imply significant changes on protein-structure or folding. Second, the effect of several mutations on the protein structure may interact, with single mutations having little effect while combined mutations having large effects on the stability of local protein folds. More research is needed to identify the real effects of mutations on protein folds and structures.

### Differential coevolution among Chlamydiae GroEL proteins

**Figure 2:**
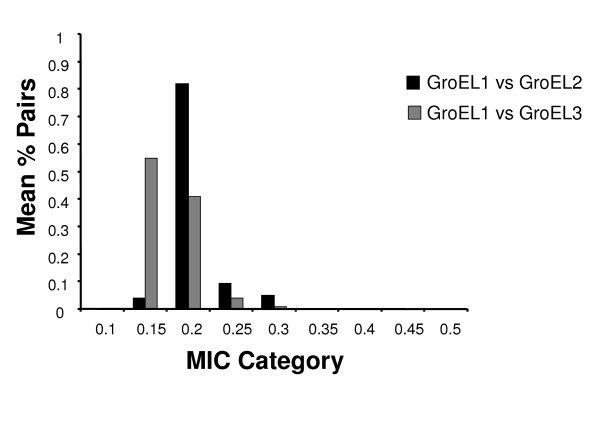
**Coevolution analysis of GroEL protein with its paralogs in *Chlamydiae***. We used the mutual information criterion (MIC) to compare the coevolution of GroEL1 vs GroEL2 (black bars) to that of GroEL1 vs GroEL3 (grey bars). We built ten MIC categories and calculated the percentage of pairs showing significant coevolution within each category (number of pairs in that MIC category divided by the total number of coevolving pairs for the comparison) in each comparison. We estimated the mean percentage of pairs per MIC category and compared both sets of data.

Functional divergence analyses detect divergence of two proteins at particular sites and evolutionary time points but do not provide a measure of the amount of decoupled evolution between the proteins after gene duplication. For example, functional divergence may have occurred between two proteins at particular sites without affecting the remaining protein sequence. In our particular case, we would not expect greater coevolution of GroEL1 with GroEL2 than with GroEL3 when averaging the coevolution parameter throughout the multiple sequence alignment. To quantify how much each of the GroEL paralogs has diverged not only functionally at particular sites but also in their evolutionary paths from GroEL1, we applied mutual information based coevolution analyses (see methods for details) between GroEL1 and GroEL2 and GroEL1 and GroEL3. Analysis of coevolution between pairs of GroEL proteins highlighted an interesting pattern that was coincident with the results of functional divergence among these proteins. We used the mutual information criterion (MIC) value as a measure of the amount of coevolution (for example, MIC ranging between zero, when sites evolve independently, and a positive value proportional to the amount of coevolution). To compare the coevolution of GroEL1 vs GroEL2 to GroEL1 vs GroEL3, we divided MIC values into 10 categories ranging between 0 < MIC > 0.5 with intervals of 0.05, estimated the proportion of sites within each category in the two sets of analyses and compared these proportions between the two coevolutionary analyses (see Methods for details). GroEL1 showed a greater mean MIC coevolution value (20% higher) with GroEL2 compared to GroEL3. In fact, most of the sites from GroEL1 coevolving with GroEL3 presented very low MIC values, indicating poor coevolution (Figure 2). In contrast, GroEL2 presented very high MIC values compared to GroEL3, indicating stronger coevolution with GroEL1 (Figure 2). In addition, most of the MIC values were above 0.2 suggesting strong coevolution. These values are in the range of MIC values obtained in previous studies examining the coevolution between amino acid sites involved in the interaction between proteins [29]. These results indicate together with the functional divergence analysis that in fact the three GroEL proteins have functionally diverged. They also indicate that GroEL2 evolved independently of GroEL1 to a certain extent but that GroEL3 show significant and more pronounced independent evolution from GroEL1 than GroEL2 does. This points to the fact that GroEL2 and especially GroEL3 may have probably evolved toward performing different regulatory mechanisms as previously suggested [15]. Their differential coevolution is also supported by data based on the analysis of the promoter regions of *groEL1*, *groEL2 *and *groEL3 *that show that *groEL2 *and *groEL3 *promoter regions in serovar D of *C. trachomatis *lack CIRCE (Controlling Inverted Repeat of Chaperone Expression) region as well as the putative σ^66 ^promoter element [30].

To further examine the selection shifts between the three GroEL protein copies we also investigated the intra-GroEL molecular coevolution and identified the differences in the coevolutionary relationships between amino acid sites among the three GroEL copies. Comparison of the coevolutionary relationships in GroEL1 to those in GroEL2 showed that while many coevolutionary relationships have been conserved in both copies (For example, amino acid pairs P217-R429, Q347-R429, Q347-P449, I348-R429, I348-P449, I348-A529, N432-P449, taking E. coli GroEL as reference sequence) other relationships have been lost in GroEL2 (D11-A404, L17-A340, L131-A340, A340-A404). Both groups of amino acid coevolving pairs include amino acid sites involved in the interaction with protein clients in the GroEL complex cavity. Interestingly, the level of coevolution between the set of pairs unique to GroEL1 (MIC = 0.225 ± 0.001) was lower than the level of coevolution for the set of pairs of sites conserved in both proteins GroEL1 and GroEL2 (MIC = 0.264 ± 0.034), indicating conservation of the main coevolutionary relationships, which are probably those highly involved in interaction with protein clients. Most interesting is the fact that GroEL3 showed no conservation of any of the intra-molecular amino acid site pairs coevolutionary relationships when compared to GroEL1 or GroEL2, thus pinpointing its unique evolutionary divergence and probable functional divergence from the other GroEL copies.

In addition, all of the *groEL2 *and *groEL3 *copies are expressed as previously shown and their non-synonymous-to-synonymous nucleotide substitutions rates ratio (ω=dNdS
 MathType@MTEF@5@5@+=feaafiart1ev1aaatCvAUfKttLearuWrP9MDH5MBPbIqV92AaeXatLxBI9gBaebbnrfifHhDYfgasaacH8akY=wiFfYdH8Gipec8Eeeu0xXdbba9frFj0=OqFfea0dXdd9vqai=hGuQ8kuc9pgc9s8qqaq=dirpe0xb9q8qiLsFr0=vr0=vr0dc8meaabaqaciaacaGaaeqabaqabeGadaaakeaaiiGacqWFjpWDcqGH9aqpdaWcaaqaaiabdsgaKnaaBaaaleaacqWGobGtaeqaaaGcbaGaemizaq2aaSbaaSqaaiabdofatbqabaaaaaaa@34EE@) indicate they are performing a distinct physiological function but that all of the *groEL *gene copies are functionally important since they are subjected to strong selective constraints (ω < 1; Table 2). These relaxed selective constraints may have occurred during the first stages after gene duplication.

**Table 2:** Analysis of selective constraints in GroEL from *Chlamydiae*. Mean replacements per non-synonymous (d_N_) sites and synonymous sites (d_S_) and the ratio between the two rates (ω) for the pairwise comparisons within GroEL1, GroEL2 and GroEL3 paralogs groups.

**GroEL group**	***d*_*S *_± SE (*d*_*S*_)**	***d*_*N *_**± **SE (*d*_*N*_)**	ω
GroEL1	0.731 ± 0.032	0.077 ± 0.006	0.105
GroEL2	0.893 ± 0.045	0.548 ± 0.027	0.636
GroEL3	0.935 ± 0.039	0.587 ± 0.025	0.628

**Figure 3:**
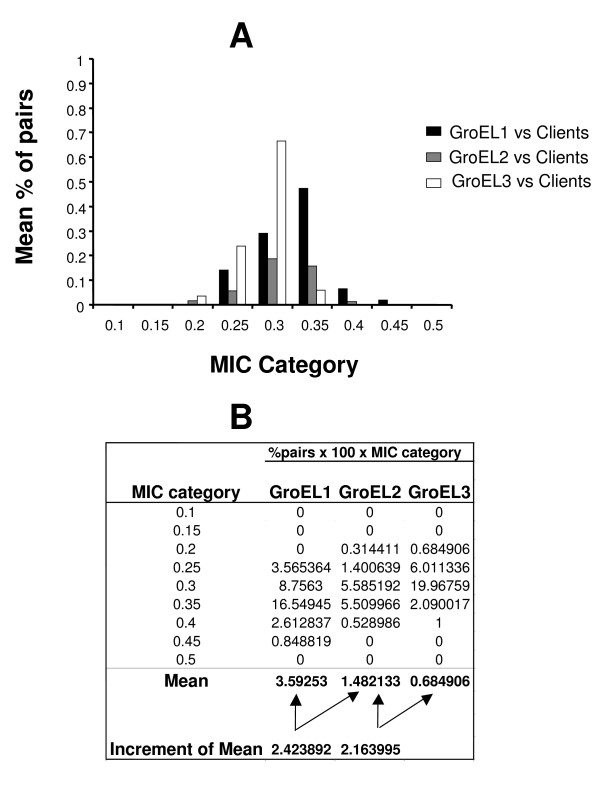
**Coevolution analyses of GroEL proteins with known protein clients for *Escherichia coli *GroEL**. We built ten MIC categories and calculated the percentage of pairs showing significant coevolution within each category (number of pairs in that MIC category divided by the total number of coevolving pairs for the comparison) in each comparison. We estimated the mean percentage of pairs per MIC category and comparison and compared the sets of data. A) Mean MIC values resulted from coevolution analyses of GroEL1 with the different protein clients (black bars), was compared to those MIC values for GroEL2 vs protein clients (grey bars) and GroEL3 vs protein clients (white bars). To better account for the distribution of MIC values in each set of coevolutionary analyses, we multiplied the % of pairs of coevolving sites in each category per 100 and per the Mean MIC value for that category and normalised the mean values obtained for all the categories in each GroEL set of comparisons as to determine the increment of coevolution between the GroEL copies and GroEL clients (B).

To investigate the difference in regulatory roles between the GroEL protein copies, we tested the strength of coevolution using the same approach as above but now between each GroEL protein copy and a set of client proteins shown to depend upon GroEL to acquire their productive functional conformation in *E. coli*. This analysis has the advantage of being relative in its interpretation because protein clients examined here are known to require GroEL to acquire productive folding and are therefore GroEL protein clients in the different bacteria examined here [31]. We were not interested in the range of new clients emerging after *groEL *gene duplication but rather in the variation of coevolution between each GroEL protein copy and the known protein clients. This test can shed some light on the question of whether functional divergence also meant divergence in the interaction patterns with each one of the different client proteins. If that was the case then we would expect that the GroEL protein copy that is more functionally divergent from the ancestral GroEL should show lower mean MIC values when tested against each protein set, indicating lower mean coevolution with these GroEL protein clients. Indeed, the percentage of sites from protein clients presenting high MIC values (MIC > 0.3) of coevolution was greater when they were tested in protein-protein coevolution analyses against GroEL1, than against GroEL2 and was higher against GroEL2 than GroEL3, coinciding with our functional divergence analyses (Figure 3A). The difference was also significant, with GroEL1 presenting an average of 2.42 times stronger coevolution with the protein clients than GroEL2, and GroEL2 presenting 2.16 times stronger coevolution with GroEL clients than GroEL3 (Figure 3B). Results hence support functional divergence between GroEL protein copies. Because of the divergence in the different coevolutionary strengths of GroEL protein paralogs and their protein clients, results also suggest that this functional divergence may have been followed by the divergence in the GroEL proteins regulatory and protein interaction networks.

### Recurrent adaptive evolution after groEL gene duplication

**Figure 4:**
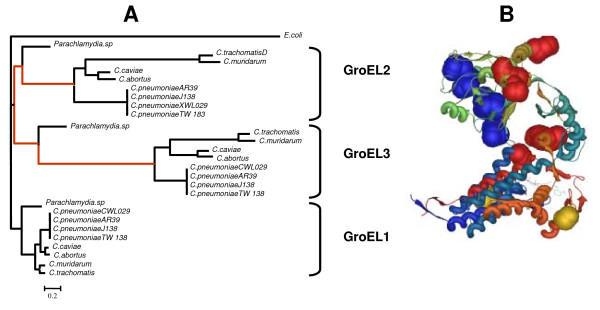
**Adaptive evolution analyses in GroEL of *Chlamydiae***. A) phylogenetic relationships between the different GroEL paralogs in Chlamydiae. The tree shows in red those branches detected to be under adaptive evolution using the maximum-likelihood free-ratio model implemented in PAML and the parsimony based approach implemented in SWAPSC. B) Three-dimensional structure of one of the *Escherichia coli *GroEL homo-tetradecamer protein structure (PDB accession number: 1SS8). Sites under adaptive evolution and functional divergence are highlighted as space-fill structures. Yellow, red and blue spheres label sites under adaptive evolution and/or functional divergence in the ATP binding/hydrolysis sites, sites pointing to the central cavity of the homo-tetradecamer GroEL complex and sites involved in substrate binding, respectively.

Here we conducted tests to further demonstrate the adaptive fixation of amino acid replacements in the GroEL protein copies after gene duplication. The rationale behind this analysis is that because of the various stages in *Chlamydiae *infections (infectious and persistent cycles) we hypothesize that the different GroEL protein copies are performing distinct functions, as their expression levels are different. We have also shown in this study that the GroEL copies are functionally divergent and that this divergence can be related to the specific ability of the different GroEL proteins to interact with the sets of proteins known to require the assistance of *E. coli *GroEL protein to acquire their productive conformation. Analysis of the sequences using the maximum-likelihood and maximum-parsimony based approaches (see methods for details) yielded very similar results that pointed to the adaptive evolution of amino acid replacements in the branches of the tree leading to GroEL2 and GroEL3 (Figure 4A). In the case of maximum-likelihood based analysis implemented in the program PAML, the model assuming different ω values for the different branches of the tree (called Free-ratio model, FRM, see Methods) was significantly better than the Goldman and Yang model (G&Y) that assumes one ω value for the entire phylogeny (LRT = 229.056; *P *< 0.001). The FRM only highlighted four branches to be under adaptive evolution (with ω values significantly greater than 1), including the two leading to GroEL2 and GroEL3, and those leading to each one of the groups to the exclusion of *Parachlamydia *species (for example, after the split separating *Parachlamydia *from *Chlamydiae *species) (Figure 4A). The parsimony based procedure implemented in the program SWAPSC also gave similar results indicating adaptive evolution after gene duplication in the branches separating *Parachlamydia *and *Chlamydiae *species and in that branch leading to GroEL3, but not in that leading to GroEL2. On average, the ω values taken from SWAPSC and PAML results for the branches under adaptive evolution ranged between (26 < ω < 264) in the branch leading to GroEL2, (2.5 < ω < 11.30) in the branch leading to GroEL3, and (1.64 < ω < 20.51) and (1.22 < ω < 18.66) after the separation between *Parachlamydia *and *Chlamydiae *species in GroEL2 and GroEL3 groups, respectively. Due to the high values of ω in the branch leading to GroEL2 group, we examined the *d*_*S *_values to determine whether these ω values were inflated due to low *d*_*S *_values estimates in PAML. Detailed examination of the *d*_*N *_and *d*_*S *_values in this branch showed that the high ω values were indeed the result of *d*_*S *_values being close to zero rather than to real increase in the fixation rate of amino acid replacements throughout the evolution of this group. We therefore could not conclude if GroEL2 was undoubtedly under adaptive evolution after the duplication leading to GroEL3 and GroEL2. Adopting a conservative view, GroEL3 was the protein showing the greatest amount of adaptive evolution, being coincident with the fact that this protein showed the greatest amount of functional divergence and decoupled evolution from GroEL1.

Examination of sites under adaptive evolution with significant posterior probabilities (PP > 0.95) identified sites involved in substrate binding and sites located in the central cavity of GroEL ring pointing toward the cavity and very probably involved in interaction with GroEL protein clients (Figure 4B). Taking all the results from the functional divergence analyses, protein-protein coevolution analyses and the adaptive evolution in each paralog group, we identified regions in the GroEL1 paralogs, GroEL2 and GroEL3, involved in interaction with proteins that have undergone changes in their selective constraints after gene duplication (Figure 4B). These results suggest that *groEL *gene duplication in *Chlamydiae *may have been followed by the GroEL paralogs' functional divergence toward acquiring different regulatory roles and establishing different protein-protein interaction network geometries.

Whether the functional divergence between the duplicated GroEL proteins meant the acquisition of completely novel functions or the subfunctionalization of the proteins copies is unclear. Placing our results into a model that supports subfunctionalization or into one that proposes neofunctionalization as the fate for gene copies after duplication requires taking into account population genetics parameters [32]. In principle, duplicated genes are lost more slowly in organisms with small effective population sizes than in those with large population sizes. The reason is that selection against harmful mutations is weaker in population with small sizes and disadvantageous mutations can drift to fixation. Gene copies resulting from gene duplication have hence more evolutionary time (opportunities) to accumulate advantageous mutations and survive despite the build up of harmful mutations. Because degenerative mutations greatly outnumber beneficial mutations the probability of neofunctionalization in small populations is rare whereas subfunctionalization is more likely to occur in these populations [33,34].

The effective population sizes of prokaryotes are considered large enough as to preclude any opportunity for subfunctionalization. However in unicellular pathogenic organisms, such as the *Chlamydiae *species analyzed in this work, their genetic effective population sizes may be greatly dependent on their multicellular hosts, which present significantly lower population sizes. In such a scenario, the genetic drift effect increases and selective constraints strength decreases, incrementing thus the probability of gene copy preservation and subfunctionalization after gene duplication. GroEL protein in *Chlamydiae *may be a striking example of such process taken to completion at the interactome level.

## Conclusion

We have demonstrated that GroEL protein copies in *Chlamydiae *species have diverged functionally after the gene duplication events. Our comprehensive bioinformatics analysis yields results that are in accordance with previously published experimental and functional data and provides further support to the divergence in the physiological and regulatory roles of the different GroEL protein copies. We also provide evidence that GroEL3 (Ct755) is more divergent from GroEL1 (Ct110) than GroEL2 (Ct604) and that this divergence was due to the fixation of amino acid replacements that modified the functional constraints in specific amino acid sites in GroEL3. Coevolution analyses performed here also support the high divergence of GroEL3 and provide further evidence that the three different GroEL copies have different interaction patterns with previously identified GroEL1 protein clients, further supporting their different regulatory roles. Finally, analysis of selective constraints supports the adaptive fixation of amino acid replacements after gene duplication mainly leading to GroEL3 and that this fixation affected functional sites involved in interaction with protein clients. Based on these analyses and conclusions we propose conducting comprehensive protein-protein interaction analyses between the different GroEL protein copies in *Chlamydiae *and the known GroEL protein clients to fully understand their functional and regulatory divergence and their role in the epidemiology, developmental and persistent stages of *Chlamydiae *infections.

## Methods

The aim of this study is to test the functional divergence between the different GroEL copies in *Chlamydiae *and provide a list of amino acid sites that may be responsible for such functional divergence, thereby detailing the functional differences among the copies. Aside from *in silico *testing of the functional divergence between the GroEL protein copies, we are interested in the quantification of such divergence and the identification of the effects of such divergence in the function of each copy. Finally, we test the effect such divergence has on the interaction of GroEL copies with previously identified GroEL-dependent protein clients [31] and we highlight the selective constraints operating in each GroEL paralog.

### Sequence alignments and phylogenetic analysis

Protein sequences coding for GroEL1 (Ct110), GroEL2 (Ct604) and GroEL3 (Ct755) were retrieved from the GeneBank database for the different species of *Chlamydiae*. The sequences, species names and the protein-coding sequence accession numbers are provided in table 1 of additional file 1. We aligned protein sequences using the program ClustalX [35] with the default settings. We then aligned nucleotide sequences concatenating triplets of nucleotides according to the multiple protein sequence alignment (alignments are available from the authors on request). Together with the *groEL *gene sequence we also obtained alignments for client proteins shown to depend on *E. coli *GroEL to acquire a productive (functional) protein conformation [31]. We obtained the sequences for each one of the *Chlamydiae *species or strains from GenBank and the accession numbers are provided in Table 2 of additional file 2. We then aligned the sequences for each one of the protein-coding genes following the same procedure detailed above.

Regarding phylogenetic analyses, for each one of the multiple sequence alignments we first used ModelTest 1.3 [36] to determine the best candidate substitution rate matrix for maximum likelihood inference. The program pinpointed TrN + I + G as first option. We used then the output generated by ModelTest as input for the program PAUP [26] and inferred a maximum-likelihood phylogenetic tree for the alignment containing the three different GroEL protein-coding sequences using the heuristic approach.

### Analysis of functional divergence

To identify amino acid replacements responsible for functional divergence between the GroEL proteins, we tested functional divergence Type I [37,38] in the multiple protein sequence alignment containing the three different GroEL copies of *Chlamydiae *after each gene duplication event. The Gu method uses a maximum-likelihood procedure to test whether there has been a significant change in the rate of evolution after gene duplication leading to the two paralogs. This method tests for functional divergence by estimating the log-likelihood value of the hypothesis assuming a value for the coefficient of functional divergence (θ > 0) and comparing this likelihood with that under the hypothesis of no functional divergence (θ = 0). Because both models are nested, they can be compared by the Likelihood-ratio test (LRT), which can be approximated to a χ^2 ^distribution with 1 degree of freedom. If the null hypothesis of no-functional divergence is rejected, the program calculates a posterior probability (PP) for a position being classified within the category of functional divergence. We established a cutoff value for the PP according to the effect that the elimination of the sets of amino acid sites having a PP value equal or higher than that cutoff value have on the θ-value test [38].

We tested functional divergence between GroEL1 and the cluster containing GroEL2 and 3, and between GroEL2 and GroEL3 using the program Diverge version 2.0 [39]. We then mapped the events of functional divergence in the phylogenetic tree including the two duplication events that gave rise to the three GroEL protein copies.

### Testing coevolution between GroEL copies

One of the questions we aimed answering was whether GroEL2 and GroEL3 diverged equally from GroEL1 or whether one of them presented less evidence for shared functions with GroEL1. A good way to test this hypothesis is by examining the coevolutionary patterns between the different GroEL copies. The stronger the coevolution between the proteins the greater would be the amount of shared evolutionary pattern and thus the greater the likelihood of sharing more functions. To test the hypothesis of coevolution between proteins we used the non-parametric method based on the mutual information criterion (MIC) developed by Korber and colleagues [40]. The mutual information is represented by the entropies that involve the joint probability distribution, P(s_i_, s'_j_), of occurrence of symbol *i *at position *s *and *j *at position *s' *of the multiple sequence alignment. The MIC values generated range between 0, indicating independent evolution, and a positive value whose magnitude depends on the amount of covariation. Variable positions included in the alignment and considered in the coevolutionary analyses were those parsimony-informative (i.e. they contain at least two types of amino acids and at least two of them occur with a minimum frequency of two). The significance of the MIC values was assessed by randomization of pairs of sites in the alignment, calculation of their MIC values and comparison of the real values with the distribution of one million randomly sampled values. To correct for multiple non-independent tests we implemented the step-down permutation procedure and corrected the probabilities accordingly [31]. MICK is implemented in the program PECA (Available from the corresponding author on request).

### Testing for protein-protein interaction divergence between GroEL copies and protein clients

One of the hypotheses we wanted to test was whether functional divergence between the different GroEL copies also involved a divergence in their coevolutionary patterns with known GroEL protein clients. To test this hypothesis we analysed the coevolution of each GroEL copy with each one of the known GroEL protein clients using the methodology described in the previous section. The strength of the coevolutionary pattern was calculated by classifying significant MIC values into the categories (0.1, 0.15, 0.20, 0.25, 0.30, 0.35, 0.40, 0.45, 0.50, MIC > 0.50). Here 0.1 included all those pairs of amino acid sites with MIC values 0 < MIC ≤ 0.1; 0.15 would include 0.1 < MIC ≤ 0.15, and so on and so forth. This categorization of MIC values allows the direct comparison of the coevolutionary results between different pairs of proteins regardless the set of MIC values obtained in each analysis. To quantify the contribution of each category to the overall MIC value, we first counted the number of pairs of sites showing MIC values within that category. We then calculated the percentage of pairs of sites included in that category by dividing the number of sites in the category by the total number of pairs of sites detected as coevolving significantly. This way, the contribution of each MIC category between pairs of proteins is comparable.

### Analysis of selective constraints

The final step in the analysis of functional divergence is the mapping of selective constraints in the protein structure after each duplication event. Here we tested whether functional divergence was the result of the adaptive fixation of amino acid replacements at functional protein regions in GroEL copies. To test this hypothesis we applied two methodologies. First, we applied a sliding-window parsimony-based approach to detect selective constraints in protein-coding genes [41], implemented in the program SWAPSC version 1.0 [42]. Briefly, the program slides a statistically optimum window size along the sequence alignment to detect selective constraints and estimates the probability of replacements per non-synonymous sites (*d*_*N*_) and substitutions per synonymous sites (*d*_*S*_). The window size is optimized by means of using a number of simulated data sets. The standard way to measure the intensity of selection when analysing DNA variability is by comparing *d*_*S *_to *d*_*N *_[43,44]. The ratio between the two rates (ω=dNdS
 MathType@MTEF@5@5@+=feaafiart1ev1aaatCvAUfKttLearuWrP9MDH5MBPbIqV92AaeXatLxBI9gBaebbnrfifHhDYfgasaacH8akY=wiFfYdH8Gipec8Eeeu0xXdbba9frFj0=OqFfea0dXdd9vqai=hGuQ8kuc9pgc9s8qqaq=dirpe0xb9q8qiLsFr0=vr0=vr0dc8meaabaqaciaacaGaaeqabaqabeGadaaakeaaiiGacqWFjpWDcqGH9aqpdaWcaaqaaiabdsgaKnaaBaaaleaacqWGobGtaeqaaaGcbaGaemizaq2aaSbaaSqaaiabdofatbqabaaaaaaa@34EE@) helps to elucidate if the gene has been fixing amino acid replacements neutrally (ω = 1), replacements have been removed by purifying selection (ω < 1), or mutations have been fixed by adaptive evolution (ω > 1). It has been shown, however, that ω is a poor indicator of the action of adaptive evolution due to the fact that signals of adaptive evolution may be swamped in the background of purifying selection under which the protein has evolved most over its evolutionary time [44].

SWAPSC uses ω to estimate the intensity of selection acting on a protein-coding region at particular branches of the tree. We used 1000 simulated data sets in our analysis obtained using the program Evolver from the PAML package [36]. To perform the simulations we took as initial parameters the average ω value, transition-to-transversion rates and codon table generated under the Goldman and Yang model, using the real sequence alignment as input. The program then slides the window along the real sequence alignment and estimates *d*_*N *_and *d*_*S *_by the Li's method. The program determines significance of these estimates under a Poisson distribution of nucleotide substitutions along the alignment.

In addition we tested adaptive evolution using the maximum-likelihood based approach implemented in the program PAML v3.15 (Yang 1997). We then compared the log-likelihood value of a model (Goldman and Yang model, hereon called G&Y) [45] that assumes one ω for the whole alignment and phylogenetic tree to a model that estimates an ω value for each branch of the phylogenetic tree (hereon called the free-ratio model FRM). We compared both likelihood values using the Likelihood ratio test (LRT) with the degrees of freedom being the number of branches in the tree minus 1.

## Authors' contributions

D.M performed the functional divergence analyses and the selective constraints analyses. M.A.F conceived the work, designed the computational analyses, conducted the analyses of coevolution and wrote the manuscript.

## Supplementary Material

Additional file 1 **Accession numbers for the *groEL *genes used in the study. **The first column collects the name of species and serovar used, the second column highlights the corresponding *groEL *gene copy, the third column accounts for the accession number of the genome to which that sequence belongs and the last columns provides the accession number of the protein corresponding to that gene. Click here for file

Additional file 2 **Accession numbers for the proteins interacting with GroEL in *Escherichia coli***. First column provides the name of the gene and the second column accounts for the SwisProt protein accession numbers. Click here for file
