# Supplementary material for: In silico identification of functional divergence between the multiple groEL gene paralogs in Chlamydiae
Source: BMC Evol Biol. 2007 May 22;7:81. doi: 10.1186/1471-2148-7-81 (PMC1892554; doi:10.1186/1471-2148-7-81)
Supplement: Additional file 1 — Accession numbers for the groEL genes used in the study. The first column collects the name of species and serovar used, the second column highlights the corresponding groEL gene copy, the third column accounts for the accession number of the genome to which that sequence belongs and the last columns provides the accession number of the protein corresponding to that gene. [file 1471-2148-7-81-S1.doc]

| **Species** | **Gene** | **Genome Acc. Num.** | **Protein Acc. Number.** |
| --- | --- | --- | --- |
| *Chlamydia trachomatis D/UW-3/CX* | GroEL1 | NC_000117 | NP_219613 |
| *Chlamydia trachomatis D/UW-3/CX* | GroEL2 | NC_000117 | NP_220120 |
| *Chlamydia trachomatis D/UW-3/CX* | GroEL3 | NC_000117 | NP_220274 |
| *Chlamydophila pneumoniae CWL029* | GroEL1 | NC_000922 | NP_224342 |
| *Chlamydophila pneumoniae CWL029* | GroEL2 | NC_000922 | NP_224972 |
| *Chlamydophila pneumoniae CWL029* | GroEL3 | NC_000922 | NP_225093 |
| *Chlamydophila pneumoniae AR39* | GroEL1 | NC_002179 | NP_445180 |
| *Chlamydophila pneumoniae AR39* | GroEL2 | NC_002179 | NP_445632 |
| *Chlamydophila pneumoniae AR39* | GroEL3 | NC_002179 | NP_445505 |
| *Chlamydophila pneumoniae J138* | GroEL1 | NC_002419 | NP_300193 |
| *Chlamydophila pneumoniae J138* | GroEL2 | NC_002419 | NP_300834 |
| *Chlamydophila pneumoniae J138* | GroEL3 | NC_002419 | NP_300955 |
| *Chlamydia muridarum Nigg* | GroEL1 | NC_002620 | NP_296764 |
| *Chlamydia muridarum Nigg* | GroEL2 | NC_002620 | NP_297266 |
| *Chlamydia muridarum Nigg* | GroEL3 | NC_002620 | NP_296515 |
| *Chlamydophila caviae GPIC* | GroEL1 | NC_003361 | NP_829507 |
| *Chlamydophila caviae GPIC* | GroEL2 | NC_003361 | NP_829841 |
| *Chlamydophila caviae GPIC* | GroEL3 | NC_003361 | NP_829733 |
| *Chlamydophila abortus S26/3* | GroEL1 | NC_004552 | YP_220012 |
| *Chlamydophila abortus S26/3* | GroEL2 | NC_004552 | YP_220334 |
| *Chlamydophila abortus S26/3* | GroEL3 | NC_004552 | YP_220224 |
| *Chlamydophila pneumoniae TW – 183* | GroEL1 | NC_005043 | NP_876411 |
| *Chlamydophila pneumoniae TW – 183* | GroEL2 | NC_005043 | NP_877077 |
| *Chlamydophila pneumoniae TW – 183* | GroEL3 | NC_005043 | NP_877201 |
| *Parachlamydia sp. UWE25* | GroEL1 | NC_005861 | YP_008179 |
| *Parachlamydia sp. UWE25* | GroEL2 | NC_005861 | YP_007029 |
| *Parachlamydia sp. UWE25* | GroEL3 | NC_005861 | YP_008258 |
