# Supplementary material for: In silico identification of functional divergence between the multiple groEL gene paralogs in Chlamydiae
Source: BMC Evol Biol. 2007 May 22;7:81. doi: 10.1186/1471-2148-7-81 (PMC1892554; doi:10.1186/1471-2148-7-81)
Supplement: Additional file 2 — Accession numbers for the proteins interacting with GroEL in Escherichia coli. First column provides the name of the gene and the second column accounts for the SwisProt protein accession numbers. [file 1471-2148-7-81-S2.doc]

| **SwissProt Entry Name** | SwissProt Accession Name |
| --- | --- |
| *glcc* | P52072 |
| *ycby* | P75864 |
| *ltae* | P75823 |
| *alf1* | P71295 |
| *nagz* | P75949 |
| *yneb* | P76143 |
| *yqab* | P77475 |
| *end4* | P12638 |
| *biof* | P12998 |
| *alr2* | P29012 |
| *his7* | P06987 |
| *fucr* | P11554 |
| *rsmd* | P42596 |
| *nana* | P06995 |
| *tldd* | P46473 |
| *thik* | P21151 |
| *ampa* | P11648 |
| *pepq* | P21165 |
| *yajo* | P77735 |
| *rluc* | P23851 |
| *pmba* | P24231 |
| *icia* | P24194 |
| *phsm* | P00490 |
| *dead* | P23304 |
| *yqji* | Q46872 |
| *xyla* | P00944 |
| *eutb* | P19635 |
| *rhle* | P25888 |
| *lipa* | P25845 |
| *ubic* | P26602 |
| *trma* | P23003 |
| *phol* | P77349 |
| *csdb* | P77444 |
| *dusb* | P25717 |
| *arac* | P03021 |
| *metf* | P00394 |
| *rimj* | P09454 |
| *frda* | P00363 |
| *yhbj* | P33995 |
| *amia* | P36548 |
| *yafd* | P30865 |
| *gaty* | P37192 |
| *add* | P22333 |
| *ybak* | P37175 |
| *gatz* | P37191 |
| *trmb* | P32049 |
| *lldd* | P33232 |
| *dnaj* | P08622 |
| *ssrp* | P32052 |
| *dusc* | P33371 |
| *yfif* | P33635 |
| *araa* | P08202 |
| *yajb* | P21515 |
| *pfla* | P09374 |
| *dhsa* | P10444 |
| *rspa* | P38104 |
| *typh* | P07650 |
| *rfbc* | P37745 |
| *fabf* | P39435 |
| *yjju* | P39407 |
| *phea* | P07022 |
| *hlpa* | P11457 |
| *ycfh* | P37346 |
| *gch1* | P27511 |
| *hem2* | P15002 |
| *parc* | P20082 |
| *dapa* | P05640 |
| *ybjs* | P75821 |
| *dada* | P29011 |
| *ycfp* | P75950 |
| *trmd* | P07020 |
| *dhas* | P00353 |
| *ftse* | P10115 |
| *suhb* | P22783 |
| *rsd* | P31690 |
| *metk* | P04384 |
| *crp* | P03020 |
| *uxac* | P42607 |
| *ypt1* | P29368 |
| *yfbq* | P77727 |
| *ints* | P37326 |
| *thih* | P30140 |
| *arge* | P23908 |
| *aldb* | P37685 |
